# From Ritual Mourning to Solitary Grief: Reinterpretation of Hindu Death Rituals in India

**DOI:** 10.1177/00302228221085175

**Published:** 2022-03-31

**Authors:** Banhishikha Ghosh, Athira BK

**Affiliations:** 1 Department of Social Anthropology and Cultural Studies (ISEK), 27217 University of Zurich, Zurich, Switzerland; 2 CSSS/SSS, 28754 Jawaharlal Nehru University, New Delhi, India

**Keywords:** Hindu death rituals, COVID-19, good and bad death, stigma, rites de passage

## Abstract

This paper considers the way the outbreak of coronavirus and the subsequent lockdown has egregiously impeded the Hindu death ceremonies and mourning rituals in India. It makes a comparative analysis of how Hindu death rituals get renegotiated, modified and reinterpreted across two vastly different regions of India, both of which have their local customs. Whilst death rituals in India are contingent on the deceased’s caste, community, class, gender and age, the impediment to the major death rituals creates a central conundrum for all mourners. It results from the substitution of ‘sacred’ ritual guidelines with new ‘profane’ ones for the ‘disposal’ of deceased COVID-19 patients. Departure from many significant pre-liminal rites, specific transition rites, and post-liminal rites has eschatological, ritual and cultural ramifications. The inability to grieve in unison during a Shraddh ceremony denies mourners any scope to quell distressing feelings about mortality which serves as a source of consolation.

## Introduction

This paper attempts to understand the ramifications that the COVID-19 pandemic has on the mourning rituals of Hindu communities in two distinctive regions of Kerala and West Bengal in India. The significance of the performative character of mourning rituals is revisited where multiple actors and meanings become circumambient to each another. A nuanced analysis of death rituals reveals a dilution of ritual practices, which serve as markers of identity distinction for members of different communities. Given the perplexity, there was consistent renegotiation, alteration and reinvention of certain *rites of passage* that provide some scope for collective mourning in a changed manner and often with the use of electronic and digital media avenues. The conversations and narrative anecdotes we came across explicate that the pandemic has played an extraordinary role in negotiating, reaffirming, and reinterpreting the previously predominant forms of spatial constellations associated with death and bereavement. Such spatial constellations are moulded through a complex eclectic of interactions of human actors (medical staff, mourners, cremators, helpers and ritual specialists), technology (online orders/passes), ‘sacred’ objects (water and fire), spaces (hospitals, cremation ghat). They embody cultural codes symbolizing disintegration and loss (Heynen, 2013).

In Hinduism, cremation involves engaging with the philosophical and mystical aspects of death (Kumari, 2021). This is also because Hindus believe that actual death takes place during cremation, and it is the ‘last sacrifice’ of a person to the gods (Parry, 1994). For the Hindus, along with cremation, it is necessary to perform certain rituals to make this critical act final (Hertz, 1960; Parry, 1994). According to Inbadas (2017), despite differences between the various schools of thought, the Indian philosophical foundation largely explains the purpose of human life as to unite with the ultimate Reality, the Divine. Hence, the moment of death is an opportunity for the ultimate transformation called *Moksha*. The smoke that flows towards the sky from the pyre is seen as a symbol of release of soul and its integration with heaven. Such cremations contribute to the idea of a ‘good death’ (Davies, 2005, p. 213). Water bodies (rivers or oceans) also play a substantial role in Hindu death rituals. Manual cremations, therefore, have historically happened on *ghats* (public open-air pyres) often located beside rivers and oceans. Serving as spatial constellations, such spaces have symbolic value as ‘sacred portals’ where the cremated remains are disposed (Quinn et al., 2014). Any cremation done in perfect observance of distinct rules is also perceived to be ‘good funeral’. This is notwithstanding the fact that certain adjustment of ritual has been an ongoing process in Hindu cremations in urban India for long (Arnold, 2017). But, during the pandemic, it became impossible to cremate the body, or follow any rite perfectly. Initially, there were also countless public furies against cremation in public places forcing the Indian state to ‘bad’ manual cremations. Hence, excess COVID-19 deaths made the process mundane (Simpson, 2021), forcing different sections of the society to modify pre-existing processes of ritual mourning to impede the withering away of death rituals to counter ‘bad deaths’.

Across the world, death of close relatives in the time of the coronavirus has been stripped of the comfort of the community leading to waves of loss, grief, anxiety, panic disorder, depression and fear among people (Kumari, 2021; Lakhani & Agarwala, 2020; Maddrell, 2020; Molokhia & Waqas, 2020). Turner and Caswell (2019) have argued that lone deaths are regarded as ‘bad deaths’ even in the United Kingdom as they contravene notions of accompaniment and open awareness. Hence, dying alone became a threat to individual and collective moral reputations during the COVID-19 pandemic. As immersion of ashes is an important Hindu ritual, burning bodies in electrical crematoriums also gave rise to a new issue as ashes of different persons get mixed up (Prajapati & Bhaduri, 2019). Matej Death as a fact of life: A perspective on the badness of death (2020), however, argues that though death never harms the one who dies, it nevertheless affects the value of one’s life. The timing of death, in particular, becomes a matter of human concern. Given that any death in India does have specific meaning in the collective consciousness involving moral and social obligations (Hertz, 1960, p. 28), inappropriate and often shocking handling of dead bodies of COVID-19 patients gave rise to widespread discontent. In this context, this paper demonstrates how the pandemic brought about certain ruptures in the conventional spatial constellations that made death a ‘public event’ and differentiate between a ‘good’ and ‘bad’ death.

## Methodology

Data for this paper have been collected through documentary evidence, observation, unstructured narratives and interviews with the crematorium officials, hospital staff, priests and kin members of the deceased. This multisided ethnography is based on two state-designated sites for the practice of COVID-19 death rituals:

a) Ivor Madom crematorium near the banks of river Bharathappuzha of Palakkad district in Kerala, and
b) Dhapa Crematorium designed specifically for the disposal of COVID-19 bodies in Kolkata, West Bengal.

Simultaneously narratives of health officials, nursing and hospital staff were congregated from some Kolkata and Palakkad hospital staff. In all, we could collect the narratives of 15 respondents from both the states in between April and September 2020. Most of the interviews took place either at the site of the crematorium or near hospitals, which sent deceased patients to these crematoriums. The data collected have been substantiated through documentary evidence and newspaper reports from these states. The selection of West Bengal and Kerala as sites of this research is, however, constrained by lack of choice of researchers to move out of these locations during lockdown during April-August 2020. While Kerala could contain the virus until August 2020, it became a victim of its own success, particularly during the second wave after April 2021 (Barnagarwala, 2021). By comparison, West Bengal had a spike in COVID-19 cases during June–July 2020 and later in April-June 2021. Notwithstanding these differences, these states followed almost similar strategies in treating patients and discharging dead bodies as most Indian states followed the guidelines issued by the central government. It is, therefore, interesting to discuss their similarities.

Both the research sites used only electrical and gas crematorium as, following the WHO guidelines, manual cremation was banned by the Indian state. Ivor Madom crematorium ground is jointly owned by Thiruvilwamala Gram Panchayat and Korappath Trust. It is situated in Thiruvilwamala village falling on the border region of Palakkad and Thrissur districts. By comparison, Dhapa Crematorium is owned and managed by Kolkata Municipal Corporation (KMC). Unlike other crematoriums of the city, it is not located near the Hooghly River, a tributary of the holy Ganges. In 1995, it was constructed for the cremation of unclaimed bodies, and then a second furnace was added to cremate stray animal carcasses. This crematorium abuts on several landfill sites and is constructed where city wastes are dumped, rendering the space unconsecrated and aesthetically unpleasant. However, post the outbreak of the pandemic, the crematorium was particularly consigned to deal with bodies of deceased COVID-19 patients by the KMC. Four more furnaces were constructed within a few months to meet heavy pressure (Ray, 2021). While there was hardly any report on separate crematoriums to handle COVID-19 deaths in Kerala during our research period,^2^ Dhapar Math in West Bengal is an illustration of the formation of new spatial constellations for funeral rituals – a space formerly designated for dumping unclaimed bodies, and animal carcasses were modified into funeral parlours for COVID-19 deaths.

The following sections will elaborate on the emerging funeral practices brought in by the pandemic and interactions between social classes, religious communities, geographies, state and biomedical definitions of health and illness.

## State Directives, Biomedical Categories and the Case of a Humanitarian Rhetoric

The directions provided by the Indian state tally with the ‘universal’ criterion for defining a COVID-19 death. Hence, the use of N95 masks for the health care practitioners, use of 1% Sodium Hypochlorite Solution for cleaning the isolation areas, maintenance of negative pressure in the mortuary, disinfection of cremation grounds and a complete injunction on post-death rituals were ubiquitous in the instructions issued by all Indian states for COVID-19 death management. The sacrality of a ‘cremation ground’ defined by its ritual, spiritual, aesthetic and functional elements became encumbered by biomedical conceptions of infectivity due to such new conditions for cremation. The staff of both crematoriums told us that rigorous adherence to uniform set of guidelines involving ASHA (Accredited Social Health Activist) staff became obligatory.

While the aforementioned uniform and profane (mundane) guidelines were in effect for the first 6 months, on 16 September 2020, an amendment was made in these guidelines by both states. These revisions permitted grieving families to hold a funeral for the departed. On 24 November 2020, the Government of Kerala published a new set of guidelines that authorized the district authority to manage every situation on a case-by-case basis, balancing the family’s rights and the risk of exposure to infection. Thus, while it talks about respecting the dignity of the dead and their traditions, it also asks staff to use standard precautions while unzipping the face end of the body bag to allow relatives to see the person from a distance.

While Kerala permitted the handing over of the bodies of deceased COVID-19 patients to their kin in sealed body bags on 24th November 2020, West Bengal did it earlier following a Calcutta High Court judgement on 16th September 2020. Later, the court on 21st September ruled that not more than six close kins of a dead person be allowed to accompany the body during the funeral (Upadhyay, 2020). The matter was brought for judicial intervention by a petitioner who accused that bodies of COVID-19 patients were being disposed of by the administration unceremoniously and in an undignified manner without showing even a resemblance of respect to the mortal remains of the dead person. The court, in its judgement, said that the bodies shall be handed over to the families only if post-mortem examination is not required. The order also stated that the head of the body bag may be unzipped by the staff at the crematorium or burial ground to allow the relatives to view the deceased. Remarking that traditions and cultural aspects are implicit in the last rites of a person’s body, the judicial bench referenced Article 25 in the Indian constitution, which decreed that every person has the right to a decent funeral. The court acknowledged that ritual beliefs and *rites de passage* were not only engrained in a social structure but also had emotional and sentimental ramifications. This judgement resulted in the state government allowing a 30-min window for family members to pay their respects and allowing the deceased’s kin to collect ashes in a sealed box after writing an application to the sub-register of the crematorium. Though it was not scientifically proved that the ashes of a COVID-19 patient could also be infectious, the state officials probably wanted to avoid any risk.

Following this intervention, Girish^1^ (43), a respondent, could perform the last rites of his mother, who died in a hospital at Kolkata from COVID-19. Yet, many bereaved families were not allowed the same. At many locations in West Bengal, the officials did not let the bereaved families to see their deceased kin or cremate them. One of the reasons for such an approach is mass cremation of dead bodies by the crematorium staff. News of mass cremation of Corona dead bodies during the pick days in the first and second phases were published in all major dallies from different Indian states. Nita shared her experience in the following way:

I did not get access to my mother in the hospital when she was admitted. Later, after she died, the crematorium staff allowed me to see her from a distance during the last rites. But I don’t know if it was her since the body was completely packed in a body bag. I also didn’t collect her ashes as I didn’t know whose ashes they would give? In the electronic crematorium, bodies were lined up one after another; there was hardly any time for the crematorium workers to separate the ashes of one deceased person from another.

Differences in dead body management could be accredited not only to a lack of adherence to government guidelines, but also due to the cacophony created by the heavy inflow of COVID-19 patients. The crematoriums staff described how they struggled in the initial days to adhere to ever-emerging guidelines. Though the guidelines referred to a ‘training’ to be given to the crematorium staff, none of the staff we talked about received it. Rather than uniform training and guidelines for managing a body, the correspondence between the health officials and the crematorium staff was often incoherent. The later was often left to interpret the guidelines for themselves. Rajesh from Ivor Madom crematorium narrated:

Even the health officials are new to the guidelines. Sometimes they call me to seek my suggestions on what to do with the body. At times, I collected the bodies from the hospitals when kins were in quarantine or couldn’t travel or provide mortuary sheets. The virus has managed to sow seeds of a fear of death among everyone, including relatives and mortuary staff. At times, I have received body bags not adequately wrapped. When one is in fear, he won’t be able to do things properly …. But we have always tried to follow the government guidelines.

Satadru, a managerial staff of the Dhapa Crematorium, similarly stated:

Due to the high inflow of bodies, often we found that guidelines like putting bodies in body bags were not followed properly. We received bodies wrapped in bedsheets, and at times in black plastic waste disposal bags. Further, everchanging guidelines from different state authorities created chaos, and it was quite challenging to follow all of them with precision. My staff is overworked.

Though the Indian state imposed uniform regulations on Hindu death rituals, it was, however, not a discrete, unitary actor (Parry, 1999). Ever-changing state regulations and confusions amongst health officials led to commotion involving cremation of the bodies. Thus, for instance, the West Bengal government issued a revised protocol on 7th May 2021, allowing the kin of the deceased patient to take the body back to their native place to cremate it (Indian Express, 2021). Such newly emerging regulations demonstrate how India’s individual states have often sought a ‘humanitarian’ and cultural scope within their profane prescriptions.

Hence, on the one hand, the state governments strictly controlled access to the body, disposal of body, and performance of rituals amidst lockdown of public activities. The judiciary also got involved in determining the correct procedure to handle such death, keeping in mind the social conventions of an ethnic community. On the other hand, over a period of time, humanitarian rhetoric and cultural rights of the kins was also evoked by the officials of both states. It appears that by revising regulations related to funerals, Indian state and judiciary have tried to partially repose faith on traditional cultural practices and rituals even at the risk of spreading the pandemic.

### Withering away of Rituals? Community, Kinship Organization and a Last Goodbye

Notwithstanding gradual recognition of cultural norms and practices related to funeral of COVID-19 dead bodies, a withering away of many significant rituals did occur due to sustained lockdown, suspension of public transport and anxieties associated with COVID-19 contraction. Our interview with a woman from a village in Palakkad affirms this. Saraswathi, a woman in her early 50s from Palakkad district, explained why she could not attend the funeral of Nandan, her family friend:

He was a family friend who passed away in Saudi Arabia, and his body could not be brought here. His family resides a few kilometres away from our home. We thought of visiting them, but all local transport was suspended then, and we could not afford a private vehicle or hire a taxi to go there.

In the case of Devan, a man in his early 60s and a neighbour, who passed away during the period of ‘unlocking’ in Kerala (August 2020), Saraswathi narrated:

Since lockdown, at least one family member from each home in the neighbourhood paid a visit. So, we thought at least one of us should go. When I went there, the health workers were checking the size of the gathering. We were wearing masks and maintaining distance. Some of those who accompanied us went inside the home, but I chose not to enter. Neighbours said he had been in the hospital for a month, and his family members were tested negative in the PCR test. Still, I maintained a distance. They brought Devan’s body to the gate in an ambulance on their way from the hospital to Ivor Madom. His wife and daughter could see his face from a distance wearing Personal Protective Equipment (PPE) kits, while his son and nephew accompanied the body to the crematorium following stipulated norms. As per the norm, his son lightened the father’s pyre. Death rituals like *Kannokku* (mourning for 15 days after the death with mandatory crying of female kin twice a day), *Pela* (restrictions on cooking at home, dietary restrictions, occasional visits by the neighbours, relatives and friends), collection of ashes on the third day of the death, *Sanjayanam* (offerings given to the departed in the presence of a priest, where the ashes/bones collected in a pot will be floated in the river) and other *Sheshakriyas* (funeral rituals) were followed. The *Pathinaru Adiyantharam* (lunch being given to the kith and kin on the 16th day of death), was also held. We all cooperated as none of the family members tested positive.

In the first case, Nandan’s family was disappointed as his body could not be brought to India. Due to COVID-19 regulations, it was impossible to meet the cultural and religious requirements of a ‘decent’ cremation. Being denied the scope to perform death rituals, they decided to just conduct post-liminal rites in the form of a remembrance gathering. But the lower financial status of Saraswathi and her family constrained them from participating in the gathering, comforting the bereaved family and fulfilling their roles as community members. In the second case, Devan’s family could conduct rituals of remembrance. But the most defining factor that allowed collective mourning and performances of caste and gender roles was an RT-PCR test, a biomedical model of detecting the presence of viruses.

In the narratives of the deceased’s kin in both states, a strong sense of discontent could be observed due to the absence of a smooth transition across three stages of *rites de passage*: pre-liminal, liminal and post-liminal (Van Gennep, 1960). The inability of the kin to personally conduct pre-liminal rites like providing mortuary sheets and performing post-death rituals has become points of contestation as such duties were mostly performed by the crematorium staff. The Ivor Madom staff maintained that they seriously performed funeral rites in the absence of a male by pouring sacred objects like ghee, milk, camphor, rose water, dried vetiver and encircled the pyre with a pot chanting *mantra* (hymns). Despite such special attention, several post-liminal rites, for example, the burning of pyre after a dip in the holy river, prayers and offerings at the sanctum of *Chudalabhadrakali* (the goddess of crematoriums), collection of bones and ashes on the third day after the cremation were exceedingly compromised.

The inability to perform such post-liminal rites was also reflected in narratives from West Bengal. For example, Nandini*,* who lost her mother-in-law to COVID-19, stated:

When my mother-in-law died, I did not even get to know the time of death. Hence, we were unable to perform many rituals. I asked the hospital repeatedly about the time of her death since it is significant to determine if she had received any *dosh.*^3^ Initially, they told me that she passed away on the night of the 4^th^ day of hospitalization. Later, the nurse said that she passed away in the evening, not night. Without proper death timing, we could not determine if she had *dipad dosh* or *tripad dosh* (different kinds of death flaws require different ritual remedies), and could not perform many post-death rituals.

Girish, whose case was referred earlier, explained how he became one of the few fortunate individuals to have seen and said goodbye to his mother, and he credits this to his ‘links’ in the hospital. He stated:

The last few days before she passed away, the doctor, my cousin, told me that there was little hope. I requested him to keep me informed; I knew officials would oversee the entire cremation process once is out of the hospital. So, I had to do everything before the municipality *swargarath* (special dead-body carrier vehicle) arrived and escorted her body. When the nurse informed me about my mother death, I quickly rushed to the hospital to perform a few rituals. I wore a PPE kit, took a few *tulsi* (basil) leaves, a little sandalwood paste, and *Ganga Jal* (holy water from the Ganges). I took a new *sari* along. I put the *tulsi* leaves on her eyes, smeared sandalwood paste on her forehead, and covered her body with the *sari*. I also quickly recited the Bhagavad Gita. That is how my mother had always wished to depart.

He also described how he took a conscious decision to admit her mother to the smaller hospital. He said:

If I had admitted my mother to a fancy hospital, I might not have had the scope to visit her after admission. It was a wise decision to keep her under the personal care of a family member. I could also do a few of the last rites, before the body was taken for cremation.

The absence of the element of physicality and tangibility involved in last rites has multiple effects. One, bereaved kin often felt a sense of not having closure or catharsis through the expression of collective grief. For instance, many rituals require close kins to follow a specific diet and abstain from cooking until *Kriya* and *Shraddh* (final feast in remembrance of the person) is done. However, it was challenging to follow these rituals with the family members being quarantined or infected themselves. Further, most post-death rituals became untenable in the absence of the ashes of the deceased. While conventionally, the ritual of *Shraddh* ended the period *ausauch* (impurity), which a grieving family is subjected to, in a situation where there is a lack of mortal remains in the form of *asthi (*ashes), the progress toward the purification of a grieving family becomes impeded. As Das (1997) indicates, such situations create an opposition between life and death particularly in so far as the mourners are concerned, in terms of the notion of impurity. The symbolism of impurity serves as a metaphor for liminality (Das, 1977, p. 115), and it is a dangerous condition of limbo to be caught within the two states of being. Rituals convert deaths from an accidental contingent event capable of questioning the entire social order into part of cosmic order (Das, 1977, p. 125). This progression gets hindered in the case of most COVID-19 deaths.

Paul (2020) has noted how, despite the government regulations, bodies of many deceased COVID-19 patients in West Bengal were not cremated but merely discarded at Dhapar Math, the landfill site near the Dhapa Crematorium. At certain other times, mix-ups and the social distancing from bodies have resulted in kin cremating other people’s bodies instead of their own. In both Kerala and West Bengal, such instances of cremation of the wrong body by family members led to double stress (Douglas, 2022). Instances like return of a ‘dead’ COVID-19 patient just before the performance of his last rites are also reported by the local media. They cremated a different body and performed the post-liminal ritual of *ashouch* (15 days of impurity followed by the death of a family member). Mistrust on state health departments, hospitals and morgues often led the deceased’s family members to use their social capital and family connections to ensure that certain rituals are performed at any cost.

## From Hospital Bed to the ‘Burning Ground’: Demands, Bargains and Negotiations

The performance of last rites often led to the chain of negotiations and bargains on several levels. In such negotiations, the kin of the deceased with greater economic, social and cultural capital had the leeway to circumvent bureaucratic rules and follow sacred rituals before the final profane cremation. Such deviation explicates how pandemic also exposes our limit to usher in an inclusive society. It is equally clear that though the narratives of administrative staff in the crematoriums in Kerala and West Bengal highlight a strict adherence to stipulated regulations, the narratives of the bereaved or the ground-level crematorium staff often reflect the opposite. These narratives reflect the arbitrariness of ritual procedures which were pursued despite some prosperous kins trying to bargain and negotiate with the ground-level staff to ease the restrictions. There were, however, minor differences in the handling of the bodies due to the differential approach of the workers in both the crematoriums. Such differences could be because both states had a differential surge in COVID-19 cases.

An administrative staff of Ivor Madom crematorium said:

Be it for the crematorium staff, the health officials, or the relatives of the dead, PPE full body suit is mandatory for all, including the ambulance driver. If the relatives were accompanying the body, there was a strict rule that only four persons would attend the body, including the ambulance driver. Our foremost prerogative is always, to burn the body as early as possible, completing the funeral rites …. Unlike in the other cases, the bone/ash collecting done on the third day after the death, the rituals to be done on the seventh, 11th and 16th day after the death were not strictly followed at times, even though we used to collect ashes and kept in earthen pots. We usually charge Rs. 2000 for the procedures of cremation. In case of COVID-19 deaths, we charge an additional amount against the purchase of PPE kits for the crematorium workers. We make sure that the ashes of the unclaimed bodies remain collected and later immersed in the Ganges.

A ground-level staff at the Ivor Madom crematorium mentioned:

We try to make sure that our ground-level staff have all the necessary equipment during the cremation. To avoid any delay in cremation, a separate road was built. Many relatives do come for cremation. In many cases, they ask for the collection of ashes, which has started recently. Initially, there were no directives from the government to provide the ashes back to the families. Later, after the issue of state directives, we started doing so. This way, they can do some ceremonies post the death of their kin.

Another ground-level staff of Dhapa Crematorium stated:

We were wearing PPE kits during the first two months of the pandemic, but during June-July 2020, many of the regulations were ignored as the state machinery almost collapsed because of an upsurge of cases. During April-May 2020, bodies were brought only by hospital ambulances and state-sanctioned body carrier services. Most of the bodies we received then were properly packed and sealed. But later, the dead came to us in heaps…and were often brought by their kin. Their kin made personal arrangements to bring the bodies by hiring private vehicles and ambulances. During this period, we saw many bodies adorned with flower garlands, sandalwood paste, basil leaves. People who had money adorned their deceased kin by bribing the ambulance staff or sometimes the hospital staff.

Thus, negotiations took place as kin of the deceased attempted to perform the last rites of the deceased within the crematorium. Secondly, negotiations and circumventions also took place on a larger structural level, with many Brahmin priests denying to perform their roles in cremation. In many cases, a helping hand has been extended from a community previously proscribed from ministering the deceased – members of opposing faiths, women and individuals identifying as lower castes. Multiple reports across both the states (Chakraborty, 2021; Sen, 2021) demonstrate how members from the Dalit and Muslim community aided the bereaved kin in the performance of Hindu death rituals. While such participation generated spaces for intercommunal cooperation, in many other instances, people only helped the bereaved kin. In the case of Ivor Madom, the responsibility of conducting the final rituals of unclaimed bodies rested on the Hindu crematorium staff. It is important to note that dilution and ruptures in conventions and norms of performance of such rituals and funeral practices (specifically by members of the deceased’s religious community) cannot be owed solely to the administrative guidelines related to COVID-19, but rather to the ‘clinical and social transactions’ (Das, 2015, p. 26) of knowledge linked to the spread of the virus. Such reports explicate the interruptions and continuities arising within structures of religion, caste, gender and kinship.

Bargains and negotiations were made on a third level as the kin of the deceased often came up with demands and requests to the hospital staff and nurses. Thus, this period saw the modifications in the roles – with the priest unable to perform many of the conventionally exiting rituals – the doctors, nurses and *ayas* (nursemaids) stepped in.

In Kerala, a Hindu doctor recited *Kalima* (Islamic prayers) for a Muslim patient in death bed, a custom that is generally performed by relatives of a dying person (‘Hindu woman doctor’, 2021).

For Sukanya (a nurse in a private hospital at Kolkata), respecting the last wishes of the dying and their kin was a personal choice:

It pains me to see how patients are dying from COVID-19. The pandemic doesn’t give any scope to family members to say the final goodbye. The pain involved in such deaths gets accentuated due to the intense loneliness and isolation in which patients are subjected. I have lost my father to COVID-19 too. He was admitted in this hospital. When he died, I put a few drops of *Ganga Jal* on his dry parched lips; I did my duty as a daughter. The day he passed away, I brought a sandalwood stick, oil and a white dhoti in my bag. Quickly before his body was taken away, I put the white dhoti over his body, and put sandalwood paste on his forehead and oiled his head. I maintained all the necessary precautions as I wore masks and sanitation kits. After he passed away, whenever a family of a COVID-19 patient approached me with similar requests, I tried to honour them without compromising my duty as a nurse. Once I put a *bindi* (a decorative mark worn by married Hindu women) and *sindoor* (vermillion worn by married Hindu women) on a woman who passed away on her husband’s request. Several times I have given *Ganga Jal* to dying patients in their last moments. All these rites would have been done if they were at home and were departing towards the crematorium with their loved ones.

Parineeti, an *aya* from a Hospital in Kolkata, also held similar views. She says,

We take care of people. And in my whole career, I have never witnessed so many bad deaths every day. This is no way to die in isolation away from the warmth of loved. I feel helpless; so, I try to do everything possible from my end, if I am asked. I put *Ganga Jal* in the mouth of so many dying patients. The doctors never stopped me and others like me from fulfilling families’ requests. One patient particularly requested me to read *Bhagavad Gita* in her last moments. I knew that she had lost her consciousness, and yet, I read a few pages without disturbing other patients in critical care. There should be dignity in death.

### “There Should Be Dignity in Death”: Bad Deaths, Not Bad Funerals

It is significant to note that while a whole set of actors, that is, family members of the deceased, hospital and crematorium staff, constantly tried to adapt to processes of death, cremation, funeral rites and grief, they also followed the state regulations. They demonstrate the consistent effort of all these groups to deal with a ‘bad death’ caused due to a sudden cataclysmic event: the COVID-19 pandemic. Not only do these ‘bad deaths’ occur untimely, they also take place when the person is alone. Considering COVID-19 deaths as ‘bad deaths’, relatives try to ensure that it should not get extended to ‘bad funerals’. Rather performance of death rituals properly was treated as a modality of undoing the bad death because even people having bad deaths deserve a dignified and respectable funeral. The way nurse Parineeti interpreted COVID-19 deaths and dying in isolation as a ‘bad death’, such a feeling is significant. Scholars like Parry (1982, p. 82) and Carstairs (1957, p. 233) note how a good death can be described as one where a spiritually prepared person for death may and even have foreknowledge of the day, and even the time he will die. Parineeti’s words resonate with Parry (1982, p. 83) and Firth’s (1989, p. 69) conceptualization of bad death, as one ‘for which the deceased cannot be said to have prepared himself’, or that one where ‘he did not die his own death’. For Firth, ‘it is not the age of the victim, but the manner of dying that is diagnostic of an *akal mrtyu*’ or a sudden unforeseen death. Here, the attachment of a large majority of Indians with religion and intertwining of culture with religion (Madan, 1989) need to be kept in mind. Simultaneously, the cooperation of health professionals to respect the dying wishes of COVID-19 patients and their families have to be understood. Thomas (2018) has observed how science and religion lead to an easy coexistence within the everyday lives and practices of Indian scientists. Any attempt to universalize or homogenize the experiences of belief and unbelief against the scale of Western rationality runs the risk of neglecting the enmeshing of these categories within the complex life-worlds of Indian medical practitioners. Hence, the nurses and doctors did not regard modern medicine and Hindu death rituals as inconsistent.

However, the constant need to provide the dying person with a dignified farewell, especially on the part of family members, has led to the emergence of ‘COVID ritual brokers’, groups of men who demand cash in return for ensuring a proper ritual cremation. *Swapan* narrates the ordeal he faced with such brokers in ensuring the body of his 32-year-old brother:

When my brother passed away, I gave 21 thousand rupees to a middleman who promised to ensure that the body was properly cremated. Apart from me, our whole family was hospitalized, and I was quarantined too; so, no one could go to perform the last rites.

Such demands of financial transaction by the crematorium/hospital authorities in exchange for performing last rites, or allowing deceased relatives to see the dead body for the last time, have come up quite often in the narratives. In one instance, with hospitals being overburdened with COVID-19 patients, a deceased patient was sent to crematorium without alerting the family (Banerjee, 2020). Loiwal (2020) reported a case of a crematorium staff demanding hefty sum from relatives in lieu of performing some last rites. The hospital, where the patient was admitted, neither informed the kins about his death nor before sending the body to the cremation ground. Incidentally, in June-July 2020, when dead bodies exceeded the capacity of all crematoriums, state health authorities sent back bodies to the homes of the relatives.

In Kerala, such rackets have been reported to be operating within hospital mortuary and crematorium, with kin being charged 30,000–60,000 rupees for conducting rites as per religious beliefs by flouting the COVID-19 protocols (Ramavarman, 2021). During the second wave, in several parts of West Bengal, one could witness the emergence of COVID ritual brokers, who promised bereaved families a package of death rituals in return for vast amounts of money. However, such networks also operate in hospitals. When such incidences came to the limelight, the West Bengal government asked police to probe it and later appointed nodal officers to supervise the cremation process (Government of West Bengal, 2021). The government then offered to pay the cremation costs of all COVID-19 deaths to impede such rackets and make the process smoother.

For people like Nakul, who lost his pregnant wife and unborn child to COVID-19, the rites of grief are impeded cataclysmically, and the long wait at crematoriums only aggravate the trauma. He states:

While the state government did issue a notice to stop such rackets and took the responsibility for doing the last rites, things were different on the ground level. I had to wait for more than a day near the Dhapa crematorium to get my wife a proper funeral. I sat down for 32 hr beside my wife’s body. I also saw some people take away their deceased relatives. They made ‘managements through packages’ of their own to cremate their kin. Doesn’t the government understand that after a person dies, he/she should be cremated within the same day? If not, the body becomes a *bashi mora* (stale death). That’s why many people are resorting to taking the help of such rackets to bury the dead.

In some instances, family members sometimes even disowned the deceased by transferring the rights for disposal of the body to the State machinery (‘Relatives of COVID victims’, 2020) due to the social stigma of being associated with a bad death. As a result, many amongst the kin sought solace in performing ‘online rituals’ through telephones and publishing obituaries in newspapers and on social media. It was also apparent that due to the restrictions imposed on funeral gatherings during this period, family and friends of the deceased resorted to being ‘digital public’ (Candon, 2016) for mourning practices. At the same time, crematorium staff tried to avoid delay in the conduct of the funeral rites as number of bodies were piling despite the governments adding new facilities (Rajwi, 2021). While such practices cannot be accorded solely to the restrictions brought about by the pandemic, extensive use of such mediums was observed even after July 2021 when the reported cases of admitted and deceased patient became strikingly less in India. It appears that illness itself, not only death, in the context of COVID-19, can be seen as a ‘quasi event’ catastrophically rupturing ongoing social relations (Das, 2015). And this has happened not only in Kolkata or Palakkad. Several other locations in India also had the similar experience.

## Conclusion

This paper addresses how diversity in the performance of ‘sacred’ Hindu rituals was initially substituted by the new ‘profane’, state-mediated procedures and government guidelines for the ‘disposal’ of deceased COVID-19 patients at the beginning of the pandemic. Subsequently, through the narratives of crematorium workers, hospital staff and families of the bereaved, the paper explores how such profane death rituals were not structured within a coordinated state healthcare system but rather embedded within a system of disparate and not well-coordinated units: state health departments, government and private hospitals, and crematoriums. These narratives do not refer to any immediacy of the situation in the study areas; they rather bring out certain general trend that several locations in India have experienced. While the sudden rise in the number of COVID-19 deaths forced many Indian states including Kerala and West Bengal to increase the number of crematories, there was pressure also on the staff to expedite the process of rituals. As a corollary, the pandemic gave rise to a new culture of funeral leading to popularity of electronic cremations, swift rituals, blemished procedure, negotiations and bargains, use of economic, social and cultural capital to circumvent bureaucratic rules, and use of electronic technology to mourn death. Notwithstanding a consistent yearning for ‘good funeral’ and post-cremation death rituals, this paper shows how state, non-state actors and family members tried to exert and also modify their notions of spirituality and faith in a changed context.

## Notes

1. Names of all interlocutors are changed to protect their privacy.

2. It was because Kerala had a lower number of deaths in the first wave than West Bengal. But during the second wave, Kerala had to increase number of crematories (Rajwi, 2021).

3. *Dosh or Mrityu dosh,* meaning death flaw in Hinduism, refers to the maleficent effects that can inflict a person’s soul if he/she dies on a certain inauspicious day/time. It can be remedied by performing post-cremation *yagnas* with the help of a priest.
